# Organic-Acid-Sensitive Visual Sensor Array Based on Fenton Reagent–Phenol/Aniline for the Rapid Species and Adulteration Assessment of Baijiu

**DOI:** 10.3390/foods13132139

**Published:** 2024-07-05

**Authors:** Lei Zhang, Yaqi Liu, Zhenli Cai, Meixia Wu, Yao Fan

**Affiliations:** State Key Laboratory Breeding Base of Green Chemistry-Synthesis Technology, College of Chemical Engineering, Zhejiang University of Technology, Hangzhou 310032, China; zl1575717@sina.com (L.Z.); kltslyq@163.com (Y.L.); czl21234@163.com (Z.C.); wmx@zjut.edu.cn (M.W.)

**Keywords:** baijiu, organic-acid-sensitive, visual sensor array, species identification, adulteration assessment

## Abstract

Baijiu is an ancient, distilled spirit with a complicated brewing process, unique taste, and rich trace components. These trace components play a decisive role in the aroma, taste, and especially the quality of baijiu. In this paper, the redox reaction between the Fenton reagent and four reducing agents, including o-phenylenediamine (OPD), p-phenylenediamine (PPD), 4-aminophenol (PAP), and 2-aminophenol (OAP), was adopted to construct a four-channel visual sensor array for the rapid detection of nine kinds of common organic acids in baijiu and the identification of baijiu and its adulteration. By exploiting the color-changing fingerprint response brought by organic acids, each organic acid could be analyzed accurately when combined with an optimized variable-weighted least-squares support vector machine based on a particle swarm optimization (PSO-VWLS-SVM) model. What is more, this novel sensor also could achieve accurate semi-quantitative analysis of the mixed organic acid samples via partial least squares discriminant analysis (PLSDA). Most importantly, the sensor array could be further used for the identification of baijiu with different species through the PLSDA model and the adulteration assessment with the one-class partial least squares (OCPLS) model simultaneously.

## 1. Introduction

Baijiu is an ancient, distilled spirit with a complicated brewing process, unique taste, and rich trace components [1,2]. These trace components play a decisive role in the aroma, taste, and especially the quality of baijiu [1,3,4,5]. Among all the trace components, organic acid is one of the most important substances in baijiu, and it is the precursor substance necessary to form the carboxylic acid ester [6,7]. Excessive organic acids often make baijiu irritating and sharp, while insufficient acid content will make baijiu taste weak. The appropriate amounts of organic acid can eliminate the bitterness of baijiu and make it full and mellow. Baijiu with different aromas, grades, and species often has different contents and types of organic acids, which constitute a complex market [2,8]. Therefore, to achieve rapid and accurate identification of various types of baijiu and their adulteration, it is necessary to establish an organic-acid-sensitive visual sensor that could monitor the trace differences existing in various baijiu samples.

Traditional methods for baijiu assessment, such as chromatography [9,10,11,12], spectroscopy [13,14], e-nose [11,15,16], etc., mostly have problems such as expensive equipment, long analysis time, or relying on precision instruments. On the contrary, the visual sensor array, with the advantages of simple equipment, fast analysis speed, and cross-response, has been more and more widely employed in the accurate analysis of baijiu and its flavor compounds [17,18]. Accordingly, the visual sensor array element materials are mainly composed of chemical response inks [19], sensitive dyes [20,21,22], pH indicators [23], or nano materials [24,25,26,27,28,29]. Suslick et al. developed metalloporphyrin chemical array sensors that can specifically identify various volatile substances based on the changes in color before and after the sensitive material reacts with volatile organic compounds [30]. Li et al. developed a three-channel simple colorimetric sensor array to differentiate between four categories of Chinese base liquor (Luzhou Laojiao), nine varieties of baijiu with distinct flavor profiles, and seven types of baijiu with different geographic origins [31]. Li et al. developed a sensor array utilizing the etching of silver nanoprisms (Ag NPRs) regulated by metal ions to successfully differentiate between 16 types of Chinese baijiu [29]. Recently, Wu et al. established a colorimetric sensor array using silver nitrate and o-phenylenediamine derivatives, which was specifically designed to detect carbonyl flavor compounds. This innovative approach proved effective in distinguishing different types of Chinese baijiu [32]. However, most visual sensor arrays focused only on either baijiu identification or adulteration assessment and fail to integrate these two important functions together.

Fenton reagent is a strong oxidizing system composed of hydrogen peroxide and ferrous ions, which can oxidize organic substances such as carboxylic acids and alcohol to an inorganic state. As shown in the following reaction equation, the main oxidizing agent in the Fenton reagent is the hydroxyl radical. Fenton reagent has the following characteristics: strong oxidizing ability, fast decomposition of hydrogen peroxide into hydroxyl radicals, and high oxidation rate [33]. On the other hand, phenol and aniline compounds can be oxidized by the Fenton reagent and further show different color changes [34]. When reacted with o-phenylenediamine (OPD), p-phenylenediamine (PPD), 4-aminophenol (PAP), or 2-aminophenol (OAP), the Fenton reagent can show obvious color changes before and after the reaction. Interestingly, this color reaction could be disturbed by the presence of organic acids [35], thus presenting either a unique color rendering mode that has the potential to be utilized in the examination of various organic acids or the color fingerprint for the analysis of different baijiu samples. However, this phenomenon is rarely used in any visual sensor array system according to the available literature.

H_2_O_2_ + Fe^2+^ → Fe^3+^ + (OH)^−^ + OH·

H_2_O_2_ + Fe^3+^ → Fe^2+^ + O_2_ + 2H^+^

O_2_ + Fe^2+^ → Fe^3+^ + O_2_^−^

**Scheme 1:**
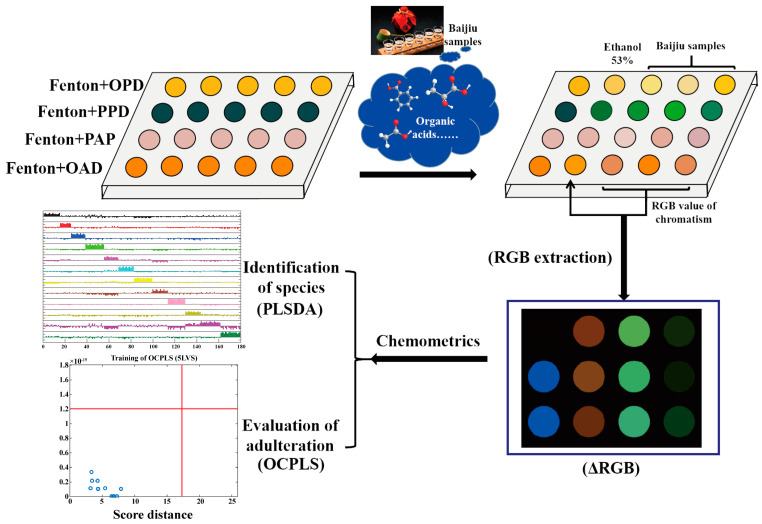
Illustration of organic-acid-sensitive Fenton reagent–phenol/aniline visual sensor array.

Herein, a four-channel organic-acid-sensitive visual sensor array based on Fenton reagent–phenol/aniline was constructed to realize the rapid detection of nine kinds of common organic acids in baijiu and the rapid species and adulteration assessment of baijiu. As shown in Scheme 1, through the fingerprint color of nine organic acids (such as acetic acid, lactic acid, hexanoic acid), either the single organic acid or the mixed organic acid samples could be quantitatively analyzed when combined with an optimized variable-weighted least-squares support vector machine based on particle swarm optimization (PSO-VWLS-SVM) [36,37] and a partial least squares discriminant analysis (PLSDA) model [38]. What is more, this novel sensor array could be used for both baijiu species identification through PLSDA and adulteration assessment through the one-class partial least squares (OCPLS) method [39]. This sensor array has excellent selectivity and sensitivity on organic acids, providing new guidance for monitoring the differences in trace organic acids in baijiu, and also has great application potential in other food industries.

## 2. Materials and Methods

### 2.1. Materials and Reagents

Anhydrous ethanol, ferrous chloride (FeCl_2_), hydrogen peroxide (H_2_O_2_), o-phenylenediamine (OPD), para-phenylenediamine (PPD), 4-aminophenol (PAP), 2-aminophenol (OAP), benzoic acid, lactic acid, isovaleric acid, hexanoic acid, heptanoic acid, acetic acid, butyric acid, isobutyric acid, and pentanoic acid were acquired from Sahn Chemical Technology Co., Ltd., Shanghai, China, and were all analytically pure. All the baijiu samples were generously supplied by Luzhou Laojiao Co., Ltd., Luzhou, China, and the detailed information is provided in Table 1.

### 2.2. Fabrication of the Organic-Acid-Sensitive Visual Sensor Array

Fenton reagent was obtained by mixing 25 mmol/L FeCl_2_ and 25 mmol/L H_2_O_2_ solutions in a 1:1 volume ratio. Then, a clean 96-well plate was prepared, and each well point received 25 μL of the freshly prepared Fenton reagent. Subsequently, 25 μL of OPD, PPD, PAP, or OAP, with a concentration of 25 mmol/L, was added as the reducing agent. The visual sensor array was composed of four channels, namely, S1, S2, S3, and S4. The composition of each channel is shown in Table S1. The images were captured using a digital camera after adding 30 μL of the samples for 5 min.

### 2.3. RGB Extraction Method

The 15-W LED plate was covered with an opaque black cloth, and a section equal to the size of the 96-well plate was precisely removed from the center, resulting in only this specific area being uniformly illuminated by a white backlight. The 96-well plate was positioned within the designated area, and photographs were captured under dark conditions using a Canon EOS 750D digital camera. The camera was equipped with a Canon EFS 18–135 mm zoom lens and securely mounted on a tripod. The images were captured using a consistent shutter speed of 1/125 s, an aperture setting of f/11, and ISO sensitivity set at 400. The camera parameters, distance between the LED panel and the camera, intensity of light emitted by the panel, and level of darkness in the indoor environment were consistently controlled to minimize any potential variations in color representation across different images. To ensure consistent color and minimize variations in the borders, Adobe Photoshop was employed to calculate the mean RGB value of a circular region (with a diameter of 25 pixels) located at the center of each well, and the RGB value matrix was obtained. The RGB value matrix refers to a 3D matrix of size m × 4 × 3, where m is the number of samples, 4 is the number of sensors, and 3 represents the R, G, and B values of the color [40]. The determination of the color vector matrix (ΔRGB) involved a comparison between the RGB values obtained from the response group and those from the control group. In addition, for the purpose of visual representation only, the RGB range was expanded to a scale of 0–255 in order to generate color differentiation diagrams. The RGB values utilized in the data analysis remained unamplified and were based on the original differences observed. All experiments were conducted in parallel, with six repetitions each, and a total of four sets of images were captured throughout the measurement process. Hence, a comprehensive dataset comprising 24 sets of parallel RGB data was utilized for color difference diagrams and chemometrics analysis.

### 2.4. Data Analysis

The MATLAB 2010a software was utilized to develop and execute the PLSDA, PSO-VWLS-SVM, and OCPLS algorithms for conducting RGB analysis.

The PLSDA [41] algorithm utilizes the simultaneous decomposition of the response matrix and extraction factor of the class matrix. By arranging the extraction factors based on their correlation, virtual vectors are encoded to represent different classes. In this encoding scheme, a value of 1 is assigned to the virtual vector *fj*, representing the *j*th element, while all other components for that class are set to 0. Each column in the response matrix corresponds to a specific class.

The procedure of PSO-VWLS-SVM model [42] can be summarized as follows: Step 1—starting with an initial set of 100 feasible solutions for the weighting vector of the variables, along with two hyperparameters (kernel widths and the relative weight assigned to the error term) in LS-SVM, a model based on LS-SVM is developed to establish a correlation between the predictor matrix and the target variable being predicted; Step 2—PSO is utilized to investigate different weighting vectors and two hyper-parameters with the aim of minimizing the prediction error, which is assessed using root square mean; consequently, the optimal combination of fingerprint variable weights for each component are obtained; Step 3—utilizing the most finely tuned variable weighting vector and hyper-parameters identified in Step 2, we construct an LS-SVM model and subsequently employ it on unfamiliar samples.

The OCPLS method [43,44] involves a specific application of partial least squares (PLS) regression between a unit vector (1, composed of ones) and feature variables without mean-centering. The estimation of PLS model complexity was conducted using Monte Carlo cross validation (MCCV) [45]. OCPLS has the capability to compute two distance measurements, specifically, the score distance (SD) determined via significant latent variables (LVs) and the absolute central residuals (ACR) of unit vector 1. Based on the computed standard deviation (SD) and average contribution ratio (ACR), a new entity will be classified into one of the following four categories: regular point (small SD and ACR); good leverage point (big SD and small ACR); class outliers (small SD and big ACR); and bad leverage point (big SD and ACR). For non-targeted analysis, regular objects are considered genuine, whereas the remaining three categories are classified as adulteration.

## 3. Results

### 3.1. The Influence of Ethanol on the Sensor Array

**Figure 1:**
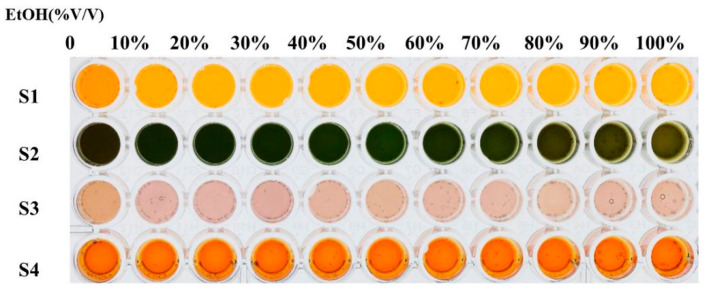
Effect of ethanol on organic-acid-sensitive Fenton reagent–phenol/aniline visual sensor array.

Since the ethanol content in commercial baijiu samples was usually between 30–60% (*v*/*v*%), the investigation of ethanol’s impact on the sensor array was imperative to guarantee response accuracy. Ten different concentrations (*v*/*v*%) of ethanol from 10% to 100% were selected for the colorimetric reaction. It can be observed in Figure 1 that when high concentrations of ethanol were present, the final color rendering of the sensor array became a little shallower than that of the pure water reaction. However, when ethanol concentrations ranged from 30% to 60%, the color rendering of the sensor array did not change significantly, indicating that the array was basically unaffected by ethanol concentration in commercial baijiu samples. Due to the fact that most of the commercial baijiu samples involved in this study owned an ethanol content of 52%, 52% ethanol was selected as the control group during the process of baijiu analysis.

### 3.2. Optimization of the Sensor Array

**Figure 2:**
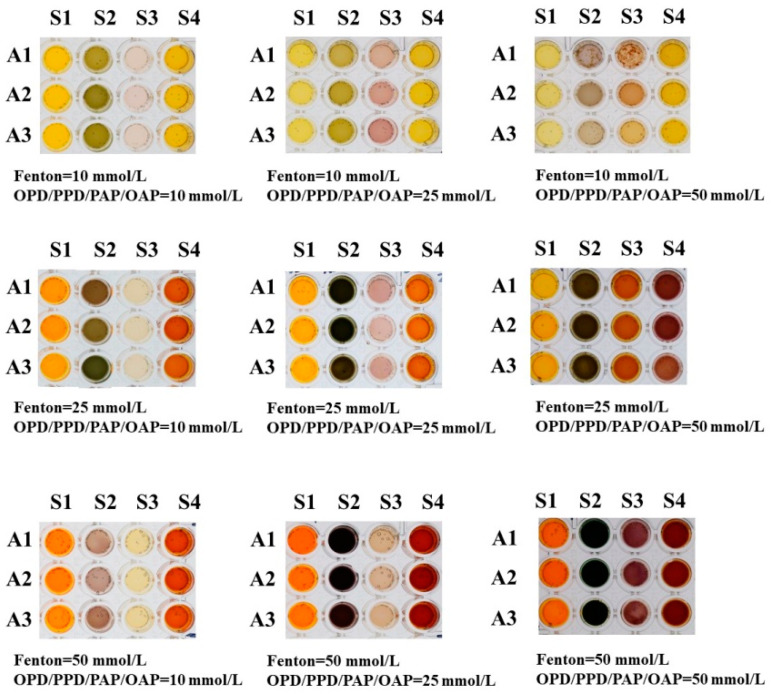
Comparison of color results under different concentrations of reactants.

**Figure 3:**
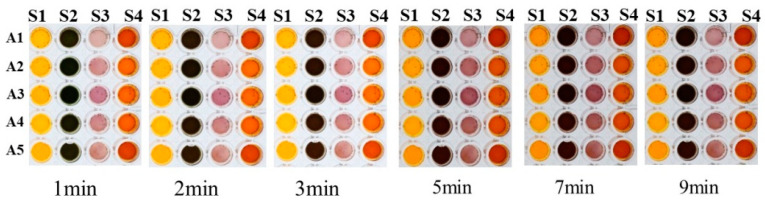
Comparison of the color results of the reaction at different times.

First, the dosage of the Fenton reagent and reducing agent in each channel was optimized, and the color rendering results after the addition of benzoic acid (A1), lactic acid (A2), or acetic acid (A3) are shown in Figure 2. When the Fenton reagent and reducing agent concentration were too large, it was easy to cause the color after the reaction to become too dark and potentially precipitate problems. On the other hand, when the concentration of the Fenton reagent and reducing agent was too small, it could lead to a light color rendering, resulting in difficulty in producing color difference responses to different organic acids. Thus, the concentration of FeCl_2_ and H_2_O_2_ used in the Fenton reagent was finally set as 25 mmol/L, while the concentrations of four reducing agents, including OPD, PPD, PAP, and OAP, were all set as 25 mmol/L to obtain the specific color difference of different acids. Subsequently, the response time of the sensor array towards organic acids was meticulously fine-tuned to achieve rapid visual analysis. The color rendering outcomes of the sensor array are displayed in Figure 3 for different reaction durations following the addition of benzoic acid (A1), lactic acid (A2), acetic acid (A3), butyric acid (A4), or isobutyric acid (A5), and the reaction time was finally selected as 5 min to obtain the best color rendering effect.

### 3.3. Color Change Response of Sensor Array to Organic Acids

**Figure 4:**
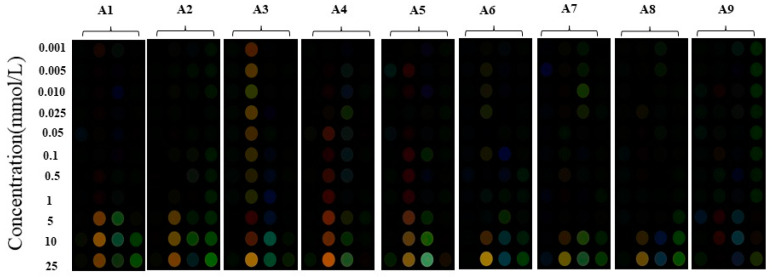
Organic-acid-sensitive Fenton reagent–phenol/aniline visual sensor array with different concentrations of organic acids added: benzoic acid (**A1**); lactic acid (**A2**); acetic acid (**A3**); butyric acid (**A4**); isobutyric acid (**A5**); valeric acid (**A6**); isovaleric acid (**A7**); hexanoic acid (**A8**); octanoic acid (**A9**).

In order to assess the sensor array’s ability to detect organic acids, a selection of nine organic acids were introduced into the array. These acids are known for their significant contribution to the overall acid content in baijiu, as well as their impact on its flavor and taste. The included organic acids are benzoic acid (A1), lactic acid (A2), acetic acid (A3), butyric acid (A4), isobutyric acid (A5), valeric acid (A6), isovaleric acid (A7), hexanoic acid (A8), and octanoic acid (A9), and the corresponding structures are shown in Figure S1. The diagrams in Figure 4 depict the color rendering and color difference of the sensor array following the introduction of various concentrations and types of organic acids. As shown in Figure 4, the utilization of a sensor array with four channels enables each organic acid to display a unique color difference diagram. Moreover, as the concentration of the organic acid increases, the sensor array gradually modifies its degree of color rendering. Consequently, this innovative sensor array possesses the ability to accurately quantify diverse types of organic acids. Different organic acids could bring about changes in the pH of the system, which will affect the reactivity of Fenton reagent to a certain extent [46]. Furthermore, organic acids could react with aniline/phenol, thus reducing the amount of aniline/phenol reacting with Fenton reagent to varying degrees, resulting in color difference [47].

In order to achieve the precise quantification of various organic acids, we employed the PSO-VWLS-SVM model for analyzing the sensor array’s RGB value matrix. Herein, the RGB value after the addition of each organic acid with various concentrations (0.001, 0.005, 0.01, 0.025, 0.05, 0.1, 0.5, 1, 5, and 10 mmol/L) was used during the quantitative analysis (10 concentrations, 24 parallel data for each concentration; in total, 240 sample data for each organic acid). The data were divided into three sets using the DUPLEX method: a training set with 120 sample data, a monitoring set with 60 sample data, and a prediction set with 60 sample data. The quantitative results are presented in Figure S2 and Table S2. The accuracy rates of the nine organic acids ranged from 96.28 ± 0.66% to 104.03 ± 0.50%, and the linear correlation coefficient (R2) was no less than 0.9998. The root mean square error of prediction (RMSEP) of all organic acids was 2.46 × 10^−6^–6.32 × 10^−5^ mmol/L. Limit of detection (LOD, 3σ) and limit of quantification (LOQ, 10σ) were determined by calculating the standard deviation (σ) of a blank sample. The results are shown in Table 2. The LOD of the nine organic acids ranges from 3.61 × 10^−5^ to 1.32 × 10^−3^ g/L, and the LOQ ranges from 1.20 × 10^−4^ to 4.38 × 10^−2^ g/L. This suggests that the sensor array has the potential to accurately analyze nine different types of organic acids in a quantitative manner.

### 3.4. Color Change Response of Sensor Array to Mixed Organic Acids

**Figure 5:**
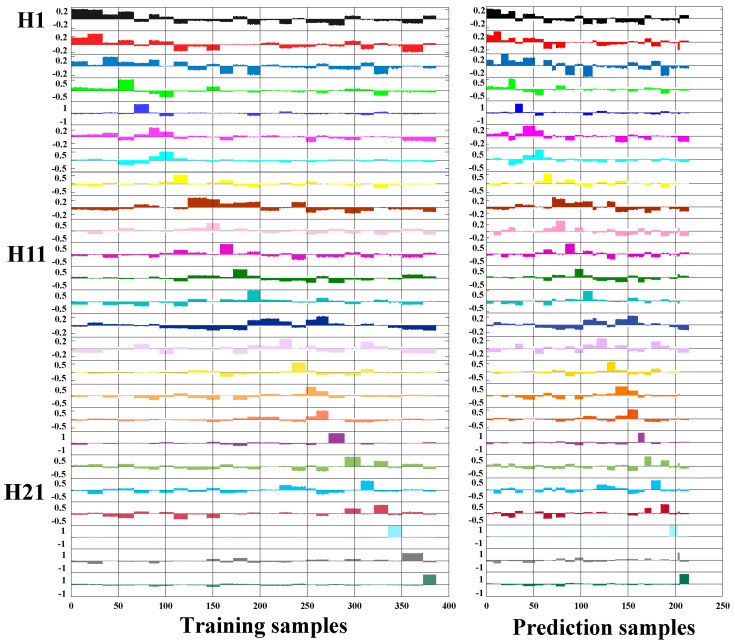
The pattern recognition results of mixed organic acids based on four-channel colorimetric sensor array via PLSDA.

As is known to all, baijiu is a mixture with multiple organic acids coexisting, so it was not enough to use the sensor array to analyze only a single kind of organic acid accurately; we also need to investigate the color rendering and analysis performance of the sensor array towards the mixed organic acids. Herein, hexanoic acid, acetic acid, and butyric acid, which are generally of a higher content in baijiu, were selected to construct the mixed organic acid samples. Since the acetic acid content in baijiu was generally stable, the concentration of acetic acid in the mixed sample was fixed as 6 mmol/L, and the concentrations of hexanoic acid and butyric acid were set as 0.1, 0.5, 1, 3, and 5 mmol/L, respectively, for mixing. Finally, 25 kinds of mixed acids were obtained, and the comprehensive particulars can be found in Table S3. The corresponding color rendering results of the sensor array following the inclusion of various mixed organic acids are depicted in Figure S3. To achieve further accurate analysis of different mixed organic acid samples, the PLSDA model was introduced to analyze the color change fingerprint information of the four-channel sensor array. The ideal quantity of latent variables was identified as nine using the leave-one-out cross-validation technique. The assigned plots of the dummy codes for all mixed organic acid samples in the PLSDA model are depicted in Figure 5, illustrating both the training set and prediction set. The dummy codes for all the samples were assigned as h1 (1, 0, 0, 0, 0, 0, 0, 0, 0, 0, 0, 0, 0, 0, 0, 0, 0, 0, 0, 0, 0, 0, 0, 0, 0), h2 (0, 1, 0, 0, 0, 0, 0, 0, 0, 0, 0, 0, 0, 0, 0, 0, 0, 0, 0, 0, 0, 0, 0, 0, 0) … h25 (0, 0, 0, 0, 0, 0, 0, 0, 0, 0, 0, 0, 0, 0, 0, 0, 0, 0, 0, 0, 0, 0, 0, 0, 1). Classification of samples from all groups was conducted based on the location of the highest dummy codes within each sample. As depicted in Figure 5, all the mixed organic acid samples from 25 groups present in both the training and prediction sets were classified into their corresponding groups and accurately recognized, and the accuracy rate, sensitivity, and specificity were all 100% (the classification results of 25 mixtures are shown in Table S4). This indicated the excellent sensitivity of the four-channel sensor array to even complex mixed organic acid coexisting samples. This result also revealed that the novel sensor array was expected to be used for the accurate assessment of different baijiu samples.

### 3.5. Selectivity of the Sensor Array

**Figure 6:**
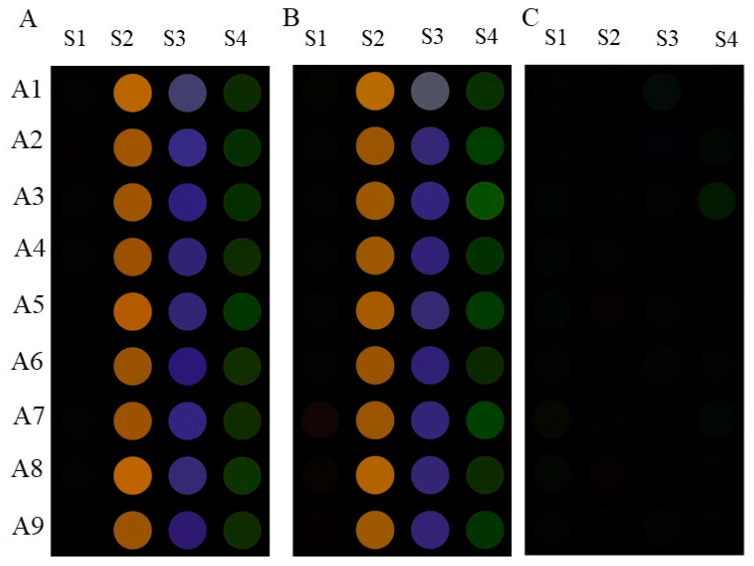
(**A**) The colorimetric results of the organic-acid-sensitive Fenton reagent–phenol/aniline visual sensor array for nine organic acids (25 mmol/L) in the presence of acetaldehyde (25 mmol/L) and ethyl caproate (25 mmol/L); (**B**) the colorimetric results of the sensor array for nine organic acids in the absence of interferences; (**C**) the color difference of the colorimetric results in the presence and absence of interference substances.

Commercial baijiu samples were always complex mixtures containing various flavor substances, such as aldehyde, ketone, ester, etc. To conduct a more in-depth analysis on the specificity of the sensor array with four channels towards organic acids, acetaldehyde and ethyl lactate, known as the two abundant flavor substances in baijiu, were selected as interfering substances and added to the system at a concentration of 25 mmol/L. Figure 6A shows the colorimetric results of the visual sensor array for nine organic acids (25 mmol/L) in the presence of acetaldehyde (25 mmol/L) and ethyl caproate (25 mmol/L). And Figure 6B shows the colorimetric results of the visual sensor array for nine organic acids in the absence of interferences. Visually, the two colors are almost identical. In order to further verify the difference in color between the presence/absence of interference, ΔRGB is shown in Figure 6C. It is evident that there was little influence on the RGB color difference, indicating that the sensor array had a good selectivity towards organic acids.

### 3.6. Identification of Various Baijiu with Different Species

**Figure 7:**
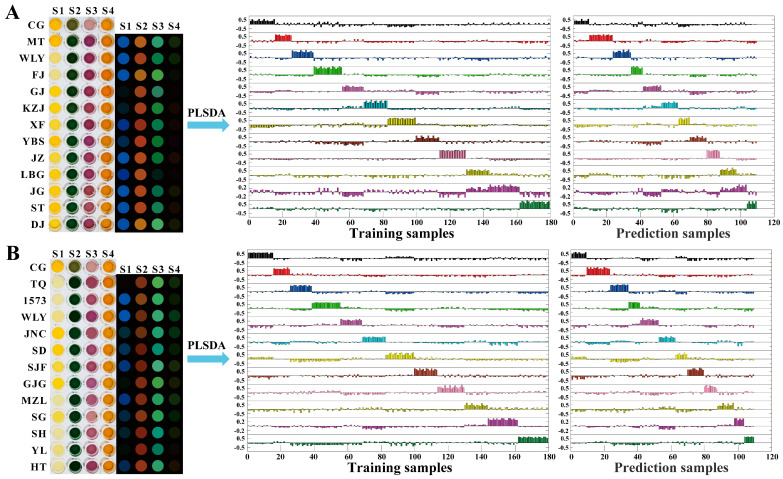
Recognition of 12 different baijiu samples with various aroma types (**A**) and species (**B**) via organic-acid-sensitive Fenton reagent–phenol/aniline visual sensor array through PLSDA.

As shown in Figure 7A, the color difference diagrams of the sensor array following the inclusion of baijiu samples were more obvious than those of the single organic acid tested above due to the higher total acid content and synergistic effect of different organic acids. Compared with the control group (CG), all 12 kinds of baijiu with various aroma types owned their unique fingerprint color change diagram. This was caused by various species and contents of organic acids in baijiu with different aroma types, which could be accurately detected by the sensor array. Furthermore, the color change fingerprint information of the baijiu samples, obtained from the four-channel sensor array, was analyzed using the PLSDA model (the detailed information of the training set and the prediction set can be found in Table S5). The ideal quantity of latent variables was identified as eight using leave-one-out cross-validation. The plots in Figure 7A depict the dummy codes assigned to the training set and the prediction set for all baijiu samples within the PLSDA model. The dummy codes for all the samples were assigned as f1 (1, 0, 0, 0, 0, 0, 0, 0, 0, 0, 0, 0), f2 (0, 1, 0, 0, 0, 0, 0, 0, 0, 0, 0, 0) … f12 (0, 0, 0, 0, 0, 0, 0, 0, 0, 0, 0, 1). As shown in Figure 7A, all the baijiu samples with different aroma types in the training and prediction sets were accurately classified into their respective groups. All baijiu samples were recognized accurately with an accuracy rate of 100%, and the sensitivity and specificity were both 100% (the classification results of 12 kinds of baijiu with various aroma types are shown in Table S6). What is more, when it came to the discrimination of 12 different baijiu samples all belonging to the strong aroma, this novel sensor array also could recognize the subtle organic acid differences in different samples. As shown in Figure 7B, 12 different baijiu samples, all belonging to the strong aroma in the training and prediction sets, were accurately classified into their respective groups, indicating that all baijiu samples were recognized accurately with the accuracy rate of 100% and the sensitivity and specificity were both 100% (the classification results of 12 different baijiu samples all belonging to the strong aroma are shown in Table S6).

### 3.7. Adulteration Assessment of Baijiu

**Figure 8:**
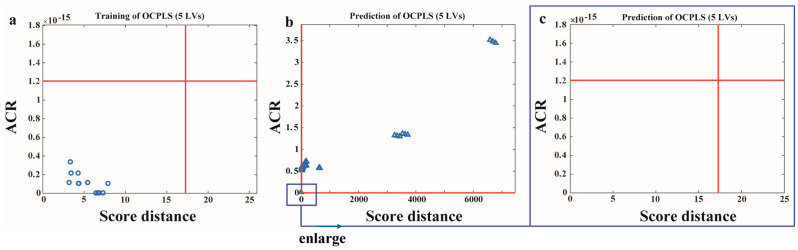
Adulteration assessment of baijiu samples via OCPLS: (**a**) the result for training samples; (**b**) the result for prediction samples; (**c**) the enlarged view of the regular point region in the result for prediction samples.

Other than different commercial baijiu samples containing organic acids with different species and contents, during the adulteration assessment processes, there was usually only a little difference in organic acid content among the different samples, which was a major challenge to the sensitivity of the sensor. The assessment performance of the sensor array for detecting baijiu adulteration was also investigated in this paper. Herein, 1573 (f1) with high a price were set as training samples, and 1573 mixed with different proportions of TQ (f2) with a low price were used as prediction samples to authenticate the functionality of the sensor array. From a financial perspective and in reality, a low level of adulteration may not yield profits, so we use 1573 mixed with different proportions of TQ (f2) (V1573:VTQ = 1:9, 2:8, 3:7, 4:6, 5:5) as the adulteration samples. The number of significant OCPLS LVs was set as five through the MCCV. Figure 8a shows the training results of pure 1573 samples. All pure samples are classified as a regular point region. This indicates that the pure samples have been accurately identified. Figure 8b shows the prediction results of adulterated TQ samples. Figure 8c is an enlarged view of the regular point region in Figure 8b. All the fraudulent samples were not assigned to the regular point region. This indicated that all adulterated samples were accurately identified, and 50–90% (*v*/*v*) of TQ adulteration could be detected. Certainly, 50% of TQ adulteration did not represent the minimum threshold for this method. On the other hand, the adulterated baijiu sample can be detected as long as the difference in organic acid content between the adulterated and genuine baijiu samples falls within the detectable range. Thus, the color change fingerprint response information provided by the four-channel sensor array through the OCPLS model can be applied to the identification of adulteration in baijiu.

## 4. Conclusions

In this paper, an organic-acid-sensitive four-channel visual sensor array based on Fenton reagent–phenol/aniline was constructed for the rapid detection of nine kinds of common organic acids in baijiu and for species and adulteration identification of baijiu. By exploiting the color-changing fingerprint response provided by the sensor array towards organic acids, single organic acid, mixed organic acids, and even the baijiu samples containing organic acid as one of the main flavor components could be accurately analyzed with the aid of chemometrics models. Furthermore, this innovative sensor has the potential not only to identify baijiu from various species but also to assess its adulteration. The excellent sensitivity of the sensor array presented in this study exhibited huge potential for practical application in the baijiu industry. Further research is needed on the simultaneous quantitative analysis of multiple mixed organic acids and the construction of an RGB matrix database for aldehydes, ketones, and acid esters in baijiu.

## Figures and Tables

**Table 1:** Detailed information on baijiu samples.

Abbr	Brand	Origin	Aroma	Alcohol (*v*/*v*%)
TQ	Luzhou Laojiao	Luzhou Sichuan	Strong aroma	52
1573	Luzhou Laojiao	Luzhou Sichuan	Strong aroma	52
WLY	Wuliangye	Yibin Sichuan	Strong aroma	52
JNC	Jiannanchun	Mianzhu Sichuan	Strong aroma	52
SD	Tuopai Shede	Suining Sichuan	Strong aroma	52
SJF	Shuijingfang	Chengdu Sichuan	Strong aroma	52
GJG	Gujigngong	Bozhou Anhui	Strong aroma	50
MZL	Yanghe	Suqian Jiangsu	Strong aroma	52
SG	Shuanggoudaqu	Suqian Jiangsu	Strong aroma	46
SH	Songhe	Luyi Henan	Strong aroma	46
YL	Yilite	Yili Xinjiang	Strong aroma	50
HT	Hetao	Bayanzhuoer	Strong aroma	36
MT	Maotai	Zunyi Guizhou	Sauce aroma	53
FJ	Fenjiu	Fenyan Shanxi	Light aroma	53
GL	Guilinsanhua	Guilin Guangxi	Rice aroma	52
KZJ	Kouzijiao	Huaibei Anhui	Mixed aroma	42
XF	Xifeng	Baoji Shaanxi	Feng aroma	52
YBS	Yubingshao	Foshan Guangdong	Chi aroma	29
JZ	Yipingjingzhi	Anqiu Shandong	Sesame aroma	52
LBG	Laobaigan	Hengshui Hebei	Laobaigan aroma	52
JG	Jiuguijiu	Jishou Hunan	Fuyu aroma	54
ST	Site	Yichun Jiangxi	Te aroma	52
DJ	Dongjiu	Zunyi Guizhou	Herbal aroma	54

**Table 2:** The linear range, quantification limit, detection limit, and relative standard deviation of nine organic acids.

Compounds	Linear Range (g/L)	LOD (g/L)	LOQ (g/L)	RSD% (*n* = 24, %)
Benzoic Acid	1.22 × 10^−2^–1.22	3.71 × 10^−4^	1.24 × 10^−3^	1.53
Lactic Acid	2.25 × 10^−3^–0.90	8.16 × 10^−5^	2.72 × 10^−4^	1.40
Acetic Acid	6.01 × 10^−5^–0.60	3.61 × 10^−5^	1.20 × 10^−4^	1.21
Butyric Acid	8.81 × 10^−3^–0.88	4.55 × 10^−4^	1.52 × 10^−3^	2.17
Isobutyric Acid	4.41 × 10^−3^–0.88	1.32 × 10^−3^	4.38 × 10^−3^	1.96
Valeric Acid	1.02 × 10^−2^–1.02	1.36 × 10^−4^	4.55 × 10^−4^	9.74
Isovaleric Acid	5.11 × 10^−3^–1.02	6.35 × 10^−4^	2.12 × 10^−3^	1.54
Hexanoic Acid	1.16 × 10^−2^–1.16	5.04 × 10^−4^	1.68 × 10^−3^	1.55
Octanoic Acid	7.21 × 10^−3^–1.44	5.12 × 10^−4^	1.71 × 10^−3^	1.41

## Data Availability

The original contributions presented in the study are included in the article/Supplementary Materials, further inquiries can be directed to the corresponding author.

## Supplementary Materials

The following supporting information can be downloaded at https://www.mdpi.com/article/10.3390/foods13132139/s1: Figure S1: Chemical structures of benzoic acid, lactic acid, acetic acid, butyric acid, isobutyric acid, valeric acid, isovaleric acid, hexanoic acid, and octanoic acid; Figure S2: Quantitative analysis results by organic-acid-sensitive Fenton reagent–phenol/aniline visual sensor array. A-I: benzoic acid, lactic acid, acetic acid, butyric acid, isobutyric acid, valeric acid, isovaleric acid, hexanoic acid, and octanoic acid; Figure S3: Color rendering results of the sensor array after the addition of various mixed organic acids; Table S1: Details of each channel in organic-acid-sensitive visual sensor array; Table S2: Quantitative results of nine organic acids provided by Fenton reagent–phenol/aniline visual sensor array (n = 2); Table S3: Detailed information of 25 mixtures prepared by mixing acetic acid, butyric acid, and hexanoic acid; Table S4: The classification results of 25 mixtures prepared by mixing acetic acid, butyric acid, and hexanoic acid; Table S5: Detailed information of baijiu samples; Table S6: The classification results of 12 kinds of baijiu with various aroma types and 12 different baijiu samples all belonging to the strong aroma; Table S7: The RGB of 12 different baijiu with various aroma types and species via organic-acid-sensitive Fenton reagent–phenol/aniline visual sensor array.
