# Supplementary material for: Organic-Acid-Sensitive Visual Sensor Array Based on Fenton Reagent–Phenol/Aniline for the Rapid Species and Adulteration Assessment of Baijiu
Source: Foods. 2024 Jul 5;13(13):2139. doi: 10.3390/foods13132139 (PMC11241830; doi:10.3390/foods13132139)
Supplement: Supplementary file 1 [file foods-13-02139-s001.zip › foods-3062098-supplementary.pdf]

Organic acid-sensitive visual sensor array based on Fenton reagent-phenol/aniline for the rapid species and adulteration assessment of Baijiu

Lei Zhang[1], Yaqi Liu[1], Zhenli Cai[1], Meixia Wu[1], Yao Fan[1,*]

[1] State Key Laboratory Breeding Base of Green Chemistry-Synthesis Technology, College of Chemical Engineering, Zhejiang University of Technology, Hangzhou 310032, China
* Correspondence: fany@zjut.edu.cn; Tel.: +86-0571-88320533

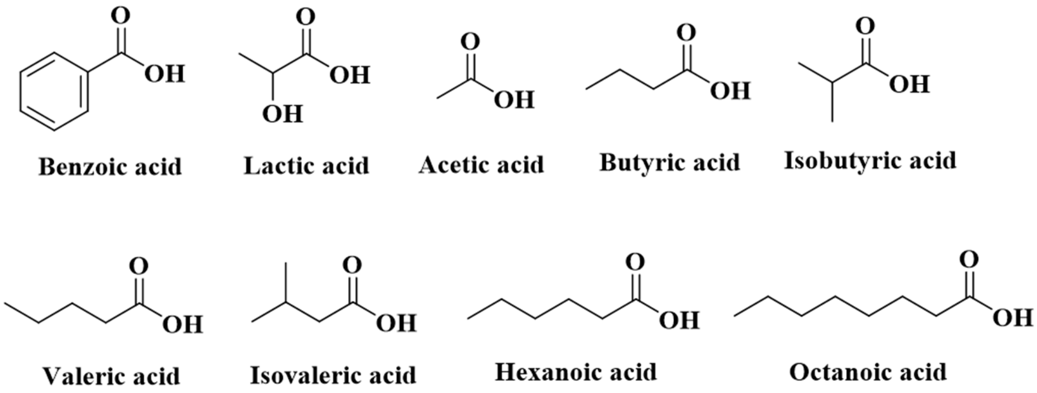

Figure S1. Chemical structures of benzoic acid, lactic acid, acetic acid, butyric acid, isobutyric acid, valeric acid, isovaleric acid, hexanoic acid and octanoic acid.

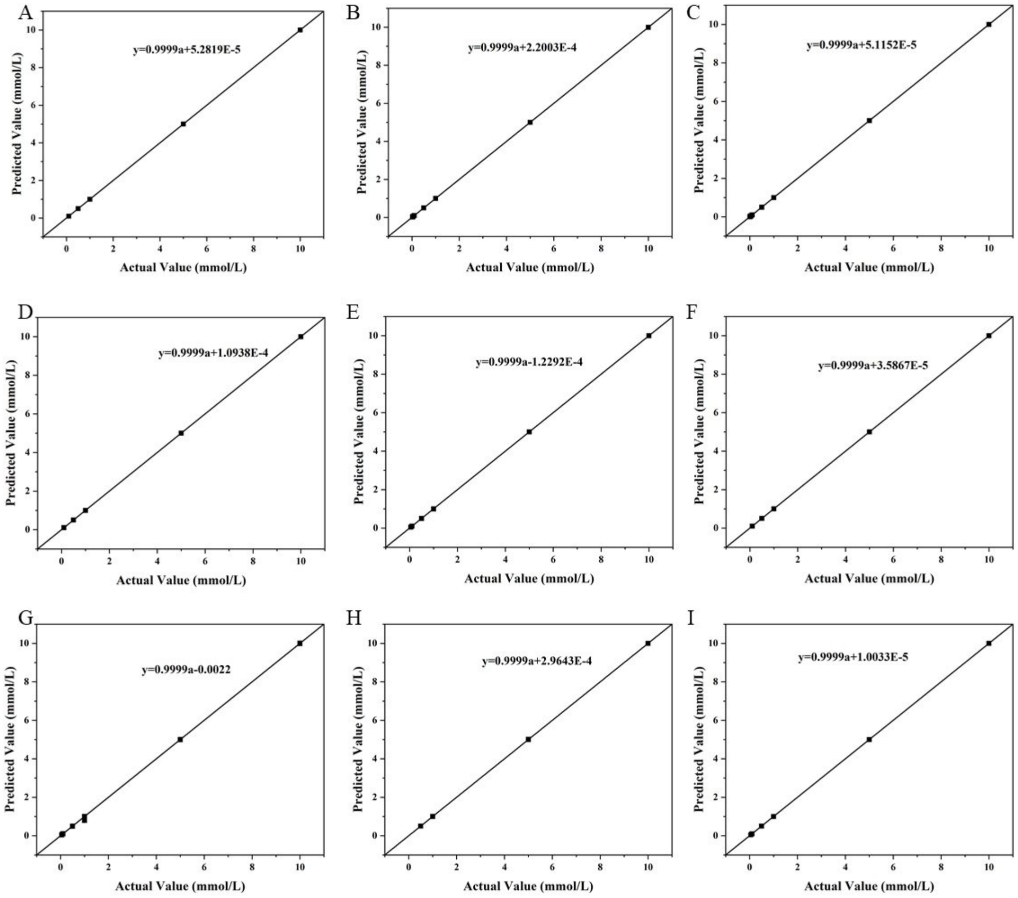

Figure S2. Quantitative analysis results by organic acid-sensitive Fenton reagent-phenol/aniline visual sensor array. A-I: benzoic acid, lactic acid, acetic acid, butyric acid, isobutyric acid, valeric acid, isovaleric acid, hexanoic acid and octanoic acid.

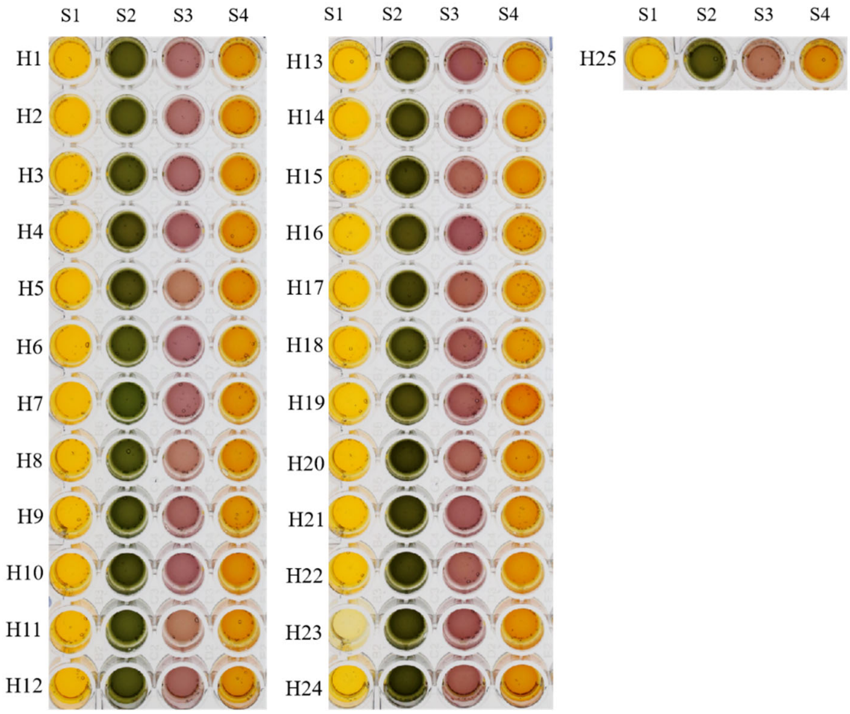

Figure S3. Color rendering results of the sensor array after the addition of various mixed organic acids.

Table S1. Details of each channel in organic acid-sensitive visual sensor array.

Channel	Composition	Amount
S1	Fe^{2+}+H_2O_2+OPD	12.5 µL, 25 mmol/L+12.5 µL, 25 mmol/L+25µL, 25 mmol/L
S2	Fe^{2+}+H_2O_2+PPD	12.5 µL, 25 mmol/L+12.5 µL, 25 mmol/L+25µL, 25 mmol/L
S3	Fe^{2+}+H_2O_2+PAP	12.5 µL, 25 mmol/L+12.5 µL, 25 mmol/L+25µL, 25 mmol/L
S4	Fe^{2+}+H_2O_2+OAP	12.5 µL, 25 mmol/L+12.5 µL, 25 mmol/L+25µL, 25 mmol/L

Table S2. Quantitative results of nine organic acids provided by Fenton reagent-phenol/aniline visual sensor array (n=24).

		**A1**	**A2**	**A3**
Training Set	R^2	0.9999	0.9998	0.9998
	RT (%)	99.92±0.12	99.68±2.65	100.12±1.13
	RMSEP (mmol/L)	2.9856×10^{-5}	2.0912×10^{-5}	1.6773×10^{-5}
Monitor Set	R^2	0.9998	0.9998	0.9998
	RT (%)	99.95±1.47	100.19±0.83	102.01±1.13
	RMSEP (mmol/L)	2.0407×10^{-5}	0.0003×10^{-3}	3.4294×10^{-5}
Prediction Set	R^2	0.9999	0.9998	0.9999
	RT (%)	101.01±0.28	96.28±0.66	104.03±0.50
	RMSEP (mmol/L)	3.8979×10^{-5}	0.0047×10^{-3}	5.8418×10^{-5}
		**A4**	**A5**	**A6**
Training Set	R^2	0.9999	0.9999	0.9999
	RT (%)	99.87±0.13	99.61±0.41	99.95±0.17
	RMSEP (mmol/L)	4.5878×10^{-5}	1.1319×10^{-5}	2.3913×10^{-5}
Monitor Set	R^2	0.9999	0.9999	0.9998
	RT (%)	99.44±1.35	99.29±0.48	100.03±0.14
	RMSEP (mmol/L)	0.1494×10^{-3}	2.0104×10^{-5}	4.7845×10^{-5}
Prediction Set	R^2	0.9998	0.9999	0.9999
	RT (%)	98.44±2.92	99.96±1.41	100.11±0.04
	RMSEP (mmol/L)	1.0005×10^{-5}	0.0013×10^{-3}	6.1318×10^{-5}
		**A7**	**A8**	**A9**
Training Set	R^2	0.9998	0.9997	0.9998
	RT (%)	98.73±1.42	99.92±1.38	99.68±2.33
	RMSEP (mmol/L)	1.2652×10^{-5}	0.0005×10^{-3}	6.3223×10^{-5}
Monitor Set	R^2	0.9999	0.9999	0.9998
	RT (%)	98.29±2.01	99.94±0.07	99.71±0.26
	RMSEP (mmol/L)	0.2460×10^{-5}	0.0019×10^{-3}	2.1852×10^{-5}
Prediction Set	R^2	0.9999	0.9998	0.9999
	RT (%)	99.73±2.70	99.84±0.29	99.68±1.16
	RMSEP (mmol/L)	1.0501×10^{-5}	0.0032×10^{-3}	3.5186×10^{-5}

RT: recovery rate; RMSEP: root mean square error of prediction; R^2: correlation coefficients deviation

Table S3. Detailed information of 25 mixtures prepared by mixing acetic acid, butyric acid and hexanoic acid.

Samples	Butyric Acid/(mmol/L)	Hexanoic Acid/(mmol/L)	Acetic Acid/(mmol/L)	Training samples	Prediction samples
H1	0.1	0.1	6	1st-17th	1st-7th
H2	0.1	0.5	6	18th-33rd	8th-15th
H3	0.1	1	6	34th-49th	16th-23rd
H4	0.1	3	6	50th-66th	24th-30th
H5	0.1	5	6	67th-82nd	31st-38th
H6	0.5	0.1	6	83rd-93rd	39th-51st
H7	0.5	0.5	6	94th-108th	52nd-60th
H8	0.5	1	6	109th-123rd	61st-69th
H9	0.5	3	6	124th-143rd	70th-73rd
H10	0.5	5	6	144th-157th	74th-83rd
H11	1	0.1	6	158th-171st	84th-93rd
H12	1	0.5	6	172nd-186th	94th-102nd
H13	1	1	6	187th-200th	103rd-112nd
H14	1	3	6	201st-220th	113rd-116th
H15	1	5	6	221st-233rd	117th-127th
H16	3	0.1	6	234th-248th	128th-136th
H17	3	0.5	6	249th-259th	137th-149th
H18	3	1	6	260th-272nd	150th-160th
H19	3	3	6	273rd-289th	161st-167th
H20	3	5	6	290th-306th	168th-174th
H21	5	0.1	6	307th-320th	175th-184th
H22	5	0.3	6	321st-335th	185th-193rd
H23	5	1	6	336th-350th	194th-202nd
H24	5	3	6	351st-372nd	203rd-204th
H25	5	5	6	373rd-386th	205th-214th

Table S4. The classification results of 25 mixtures prepared by mixing acetic acid, butyric acid and hexanoic acid.

Sample group	Training samples		Prediction samples	
	Positive	Ture Positive	Positive	Ture Positive
H1	17	17	7	7
H2	16	16	8	8
H3	16	16	8	8
H4	17	17	7	7
H5	16	16	8	8
H6	11	11	13	13
H7	15	15	9	9
H8	15	15	9	9
H9	20	20	4	4
H10	14	14	10	10
H11	14	14	10	10
H12	15	15	9	9
H13	14	14	10	10
H14	20	20	4	4
H15	13	13	9	9
H16	15	15	9	9
H17	11	11	13	13
H18	13	13	11	11
H19	17	17	7	7
H20	17	17	7	7
H21	14	14	10	10
H22	15	15	9	9
H23	15	15	9	9
H24	22	22	2	2
H25	14	14	10	10

Table S5. Detailed information of Baijiu samples.

Sample group	Training samples	Prediction samples	Sample group	Training samples	Prediction samples
MT	1st-15th	1st-9th	TQ	1st-15th	1st-9th
WLY	16th-25th	10th-23rd	1573	16th-25th	10th-23rd
FJ	26th-38th	24th-34th	WLY	26th-38th	24th-34th
GL	39th-55th	35th-41st	JNC	39th-55th	35th-41st
KZJ	56th-68th	42nd-52nd	SD	56th-68th	42nd-52nd
XF	69th-82nd	53rd-62nd	SJF	69th-82nd	53rd-62nd
YBS	83rd-99th	63rd-69th	GJG	83rd-99th	63rd-69th
JZ	100th-113th	70th-79th	MZL	100th-113th	70th-79th
LBG	114th-129th	80th-87th	SG	114th-129th	80th-87th
JG	130th-143rd	88th-97th	SH	130th-143rd	88th-97th
ST	144th-161st	98th-103rd	YL	144th-161st	98th-103rd
DJ	162nd-179th	104th-109th	HT	162nd-179th	104th-109th

Table S6. The classification results of 12 kinds of Baijiu with various aroma types and 12 different Baijiu samples all belonging to the strong aroma.

**Sample group**	**Training samples**		**Prediction samples**	
	Positive	Ture Positive	Positive	Ture Positive
MT	15	15	9	9
WLY	10	10	14	14
FJ	13	13	11	11
GL	17	17	7	7
KZJ	13	13	11	11
XF	14	14	10	10
YBS	17	17	7	7
JZ	14	14	10	10
LBG	16	16	8	8
JG	14	14	10	10
ST	22	22	4	4
DJ	18	18	6	6
TQ	15	15	9	9
1573	10	10	14	14
WLY	13	13	11	11
JNC	17	17	7	7
SD	13	13	11	11
SJF	14	14	10	10
GJG	17	17	7	7
MZL	14	14	10	10
SG	16	16	8	8
SH	14	14	10	10
YL	22	22	4	4
HT	18	18	6	6

Table S7. The RGB of 12 different baijiu with various aroma types and species by organic acid-sensitive Fenton reagent-phenol/aniline visual sensor array.

	S1	S2	S3	S4
CG	238.1952	92.1344	183.3584	215.784
	185.7856	90.328	141.5872	145.5104
	2.336	15.6624	137.872	2.096
MT	249.8944	37.2496	127.1872	202.2384
	193.2864	65.5872	55.4576	124.1872
	2.88	13.4112	95.424	1.7856
WLY	237.7552	33.1808	129.5168	204.256
	220.3216	54.2208	54.4272	131.1312
	124.6464	10.8624	84.4272	2.9808
FJ	235.4848	42.6416	131.5488	205.9584
	223.6928	65.5104	55.3856	117.064
	143.9328	14.1696	80.16	1.1984
GL	248.8112	43.1728	134.2736	210.3888
	213.8032	64.4112	60.392	121.6432
	32.8656	15.0208	81.2896	0.9904
KZJ	247.504	47.168	142.9152	206.1696
	215.736	71.32	68.232	129.8704
	44.3152	15.3904	81.28	2.7056
XF	244.0464	42.0112	128.6256	200.16
	218.1424	71.0448	55.4112	124.3344
	64.1264	16.5936	75.4976	1.1376
YBS	249.192	31.944	130.4912	200.3616
	200.2496	65.0592	59.8288	132.224
	7.1904	14.0928	99.6944	2.9168
JZ	242.5136	36.928	139.1584	200.2416
	217.1584	68.2416	65.7552	123.0992
	69.7664	16.2576	92.8928	1.4352
LBG	246.0752	31.3216	135.3664	208.552
	211.8816	62.392	60.3776	130.1552
	36.2528	15.544	95.0768	2.6672
JG	246.9056	37.8912	143.0592	203.1232
	209.7648	72.3552	70.8384	132.664
	26.2608	17.768	78.5056	3.6144
ST	248.9568	37.04	152.2224	207.24
	200.9584	71.8304	85.4752	139.6016
	7.8304	15.3408	93.0336	1.3248

Table S7. The RGB of 12 different baijiu with various aroma types and species by organic acid-sensitive Fenton reagent-phenol/aniline visual sensor array (continued).

DJ	243.0896	25.3632	134.2336	202.5008
	210.9088	63.5616	61.4992	138.5088
	43.8656	12.8512	84.9248	5.2816
TQ	232.7264	11.848	139.3136	204.744
	224.9168	43.704	67.8784	127.0352
	163.8944	6.776	85.2832	2.24
1573	233.344	21.9008	154.7872	209.1648
	225.8864	61.9296	84.3104	129.1872
	167.7056	10.9072	108.2752	3.0112
WLY	237.064	14.9536	135.3184	210.4352
	224.888	41.0768	63.4704	138.6768
	142.4208	6.0768	105.552	5.5248
JNC	245.8016	23.4912	149.224	212.5088
	204.6752	60.7552	81.7392	145.1744
	14.1152	10.0416	92.3504	4.0336
SD	248.2688	24.0112	149.8656	207.752
	212.5632	64.5408	81.2864	139.2464
	24.1648	14.672	112.3024	5.9184
SJF	241.8048	27.912	154.984	208.008
	220.1984	68.1344	88.5952	140.4528
	76.7216	14.7456	103.9616	2.76
GJG	247.7552	16.7584	154.0256	208.7488
	216.8112	63.0192	90.0672	147.8624
	40.6256	13.9232	126.2352	10.32
MZL	232.2064	15.264	154.2016	205.2064
	226.3424	56.2432	89.8912	136.1824
	164.0704	10.5872	113.6512	9.872
SG	242.048	17.0192	180.624	210.3392
	219.2192	54.1888	130.1104	144.6576
	75.2208	10.4384	127.9936	5.3616
SH	232.2288	16.8784	160.2512	206.1824
	226.12	58.536	94.768	135.6336
	168.1504	12.7232	104.7216	5.8368
YL	236.8448	18.8	152.768	206.2128
	222.5456	62.5056	85.1408	139.744
	132.2576	11.624	102.2032	6.5808
HT	231.7616	9.1504	132.2048	206.4176
	222.0208	38.4176	60.9184	136.3472
	154.2608	6.848	89.5088	4.9296
